# Specific cutaneous involvement in a child with monoblastic leukemia

**DOI:** 10.11604/pamj.2014.17.307.2726

**Published:** 2014-04-22

**Authors:** Siham Cherkaoui, Said Benchekroun

**Affiliations:** 1 Department of Hematology and Pediatric Oncology, hospital 20 Août1953, Ibn Rochd University hospital, Casablanca, Morocco

**Keywords:** Monoblastic leukemia, fine needle aspiration, splenomegaly

## Image in medicine

**Figure 1:**
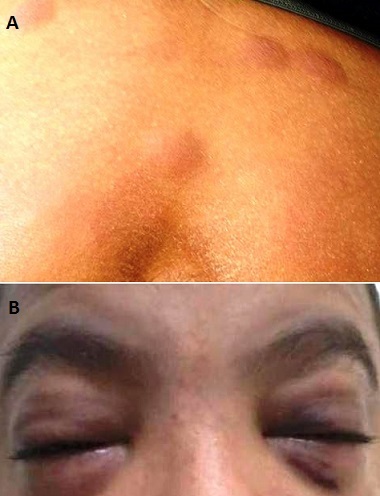
A) Cutaneous nodules; B) Facial edema

A 13-year-old boy, admitted with hemorrhagic syndrome and fever. Physical exam showed dermo-hypodermic nodular lesion on the whole body (Figure 1), facial edema (Figure 2), splenomegaly at 2 cm and angulo mandibular lymph node of 2cm. Count blood cells found white blood cell at 1360/mm3 with 94% of blasts, anemia at 8.3 g/dl and thrombocytopenia at 38000/mm3. Morphological examination of bone marrow confirmed diagnosis of acute monoblastic leukemia. Flow cytometry showed positivity of CD11c, CD13, CD33, CD34, HLA-DR. karyotype was normal. Child received chemotherapy.

